# Relationship Between Brain-Derived Neurotrofic Factor (Bdnf) and Sleep on Depression: A Critical Review

**DOI:** 10.2174/1745017901713010213

**Published:** 2017-11-21

**Authors:** Bárbara C. Monteiro, Suzana Monteiro, Maristela Candida, Nathalia Adler, Flavia Paes, Nuno Rocha, Antonio Egidio Nardi, Eric Murillo-Rodriguez, Sergio Machado

**Affiliations:** 1 Laboratory of Panic and Respiration, Institute of Psychiatry, Federal University of Rio de Janeiro, Rio de Janeiro, Brazil.; 2 Polytechnic Institute of Porto, Health School, , Portugal; 3 Laboratorio de Neurociencias Moleculares e Integrativas, Escuela de Medicina, División Ciencias de la Salud, Universidad Anáhuac Mayab, Merida, Mexico; 4 Intercontinental Neuroscience Research Group, Brazil.; 5 Physical Activity Neuroscience Laboratory (LABNAF), Physical Activity Sciences Post-Graduate Program, Salgado de Oliveira University (UNIVERSO), , Brazil

**Keywords:** BDNF, Sleep, Depression, Antidepressants, Sleep quality, BDNF studies

## Abstract

The Brain-Derived Neurotrofic Factor (BDNF) is one of the most important neurotrophins in the brain and it is suggested influences the activity of the serotonergic, noradrenergic and dopaminergic pathways. In the last few years, it has been hypothesized that BDNF level is related with depression and sleep. Several studies show that depressive subjects present low levels of BDNF in the brain. Poor sleep quality is also related with alterations in the BDNF concentration. Some authors argue that most of the cases show that impaired sleep quality increases the stress and, consequently, the vulnerability to depressive disorders, suggesting that there is a relationship between sleep, depression and BDNF levels.

## 1 INTRODUCTION

Depression is currently among the four major diseases affecting the world population and is linked to high rates of impairment and mortality [1-3], and can be defined as a disorder with heterogeneous biological bases with a chance of affecting 10% -30% of women and 7% -15% of men throughout their lives. Clinical and preclinical studies suggest that Brain-Derived Neurotrofic Factor (BNDF) expression could be involved in behavioral phenomena linked to depression, and that modulation of this neurotrophin would also mediate the action of antidepressants [4].

BDNF is the main neurotrophin in the human brain [5], playing a critical role in the development and protection of the central nervous system as well as synapse regulation, learning and memory [6, 7]. BDNF widely contributes to neuroplasticity in the human brain, namely axonal and dendritic growth and remodeling, neuronal differentiation, synaptic growth and transmission, neurotransmitters modulation and long-term potentiation [8-10]. One of the main functions of BDNF is to promote the development and stability of neuronal connections, with the hippocampus being an important site for its action [11]. It’s known that BDNF is required for the proper development and survival of GABAergic neurons, cholinergic and serotonergic antidepressants [2, 4].

The relationship among BDNF, sleep and depression has been speculated. A low neurotrophic activity is associated with a reduced number of cells in the prefrontal cortex, amygdala, and decreased hippocampal size, indicating that BDNF may play an important role in the development of depression [12]. Some authors speculate that BDNF has a potential role in the pathology and treatment of numerous psychiatric disorders [2]. Studies showed low BDNF levels in un-medicated depressed individuals, but without association with disorder severity [13]. BDNF gene is an important candidate in the discovery of the action mechanisms of antidepressants, since BDNF plays an important role in the functioning of the serotoninergic system [14]. The relationship between antidepressants and BDNF in rats has shown that the administration of different types of antidepressants increases the levels of BDNF production in the hippocampus [14], but this effect can been see only with nonpeptidic agonists [15]. Antidepressants that decrease BDNF levels can be effective in treatment of depression, however can alter sleep pattern [16], which may increase GABA_A_Rs on the membrane of excitatory cortical neurons, while increase of slow wave sleep can return to baseline levels [17], while adenosine A_1_ receptors (A_1_R) plays a decisive role for antidepressant effects on sleep deprivation [18].

The relationship between depression and sleep disturbance is well delimited, but while typical depression is usually accompanied by insomnia, atypical depression is usually related to symptoms of hypersomnia [19]. It has been speculating the relationship between BDNF, sleep and depression. There is evidence that BDNF levels influence sleep patterns in individuals with depression [12]. Some authors postulate that increasing BDNF levels may improve the sleep quality of depressed subjects, and in these cases the BDNF levels make a difference in the results found in treatment. The impact of increasing BDNF on sleep will be discussed later. Thus, the purpose of this article is to trace the relationship between BDNF and insomnia in depression and what the possible result of the interaction of these factors [19]. With respect to the neurobiology of depression, many authors consider that one of the main triggers of the disorder is stress. Thus, the BDNF hypothesis in depression, postulates that stress reduces the concentration of BDNF in structures of the limbic system, responsible for the emotions [17]. Therefore, the comprehension about this possible relationship could support better decision making for treatments, for example, choosing the more appropriate antidepressant to be used for a certain patient. Although few studies appointing to a relationship among BDNF levels, depression and sleep, this issue is still unclear.

## 2 BDNF AND SLEEP

Sleep can be defined as “...a recurring, reversible neuro-behavioral state of relative perceptual disengagement from and unresponsiveness to the environment. Sleep is typically accompanied (in humans) by postural recumbence, behavioral quiescence, and closed eyes...”. The sleep-wake cycle involves neurophysiological states like wakefulness, slow wave sleep and rapid eye movement sleep [17]^.^ The relationship between duration, efficiency, and timing are crucial to understand sleep, and alterations in this factor may lead to sleep disorders [19, 20]^.^

Sleep disturbances have been characterized in terms of abnormalities in alertness/sleepiness, continuity or efficiency, duration and timing. According with International Classification of Sleep Disorders the most common sleep disorders are: Dyssomnias, Parasomnias, Sleep Disorders Associated with Mental, Neurologic, or Other Medical Disorders and Proposed Sleep Disorders [21]. Insomnia and hypersomnia are the most frequent sleep disorders in population [22, 23]. The management of these two sleep disturbances includes several therapeutically approaches, such as pharmacological treatments [24-26].

Sleep impairment may lead to severe physical and mental problems, as sleep deprivation is usually followed by enhanced vulnerability to stress which can decrease BDNF production. It’s known that serum BDNF levels are associated to sleep, even in patients who do not have sleep problems [27, 28]^.^ In a study with 126 patients with MDD, was found that increased BDFN levels has been associated with N-REM sleep improvement and enhanced slow wave activity, but on the other hand, reported no relationship between BDFN and insomnia improvement. It was found that the reduction of BDFN levels is associated with improvement in hypersomnia [16].

Giese and cols investigated if stress levels influence the association between sleep and BDNF levels, and found that the sleep quality interfere directly in the relation of stress with BDNF levels [16]. In another study they investigated BDNF serum levels in 26 patients with insomnia and compared the results with a control group and found that insomniacs present decreased serum BDNF levels, when compared to control group [28]. Other study examined 44 insomniacs and found that sleep problems are related to impaired BDNF synthesis and that improvement in sleep patterns leads to enhance quality of life [28] (Table **1**).

## 3 BDNF AND DEPRESSION

Research has evidenced the link of BDNF as a molecular factor involved in the pathophysiology of mental disorders. Low levels of this factor are directly linked to major depressive disorder [29]. Studies have shown that infusion of BDNF into the brains of animals produces an antidepressant effect [30, 21]. In addition to this action, BDNF promotes the functioning and expansion of serotonergic neurons in the brains of adult rats [11]. The administration of BDNF produces an antidepressant effect in two animal models of depression. According to the study, infusion of BDNF near the PAG and raphe nucleus allows the factor to have access to a greater number of serotonergic cells. According with the authors, experiments in the infusion of BDNF into rat brain show an increase in activity in the serotonergic, noradrenergic and dopaminergic pathways of numerous brain areas such as cortex, hippocampus, striatum and nucleus accumbens [29]. To reinforce this point of view, some studies show that at the time of necropsy, a greater expression of BDNF in the hippocampus of individuals treated with antidepressants when compared to untreated depressants [32,33,34].

Hippocampus is a brain region designated as one of the BDNF foci, and studies aiming to assess the level of neurotrophin in depressed patients generally turn their attention to this site. Clinical and preclinical studies point to a loss of the total volume of neurons in the Hippocampus of adults with depression. In the pathophysiological basis of the disease we have the question of neuronal plasticity and it is now well established that BDNF plays an important role in neuronal plasticity and maintenance [33].

Animal studies show that the use of agomelatine, an antidepressant drug, for the treatment of depressive disorders leads to an increase in BDNF expression in the prefrontal cortex and in the hippocampus [5]. This fact reinforces the hypothesis of the BDNF x depression relationship, since the use of antidepressant would improve the expression of the neurotrophic factor. Non-drug treatments for depression, such as Electroconvulsive Therapy (ECT), also generate an increase in BDNF expression in the brain, which further underscores the linkage of BDNF levels with depressive disorders [35].

Increased levels of BDNF protein in the brain are consistent with the efficacy of MAO inhibitors and electroconvulsive therapy and may be a predictor of the antidepressant action of interventions that are more effective in the treatment of major depression. All the evidence pointing to the fact that treatment for depression shows an improvement in BDNF expression, it is still unclear how discontinuation of antidepressant medication would affect these levels [35]. Thus, BDNF levels are apparently positively regulated in humans through antidepressant treatments [2, 36] (Table **2**).

## 4 HYPOTHETICAL RELATIONSHIP BETWEEN BDNF AND SLEEP ON DEPRESSION

In most of cases, Major Depressive Disorder is accompanied by complaints about sleep quality. Sleep disturbance is one of the main residual symptoms after treatment with antidepressants and the main risk factor for relapse in major depression who are in remission [18] Some biomarkers present in the development of depression and response to treatment are linked to sleep quality [16].

Studies that observe the relationship of the effect of agomelatine with BDNF levels show that the effect that the drug generates on the circadian rhythm would be a consequence of the increase of BDNF levels, since the neurotrophin expression is regulated by the light-dark cycle, both in rats, and in humans [5].

There is a hypothesis that not all antidepressants generate an improvement in the serum BDNF level, like bupropion [16], and the improvement of this neurotrophin does not coincide with an improvement in the clinical symptoms of depression. According to some results found, changes in BDNF concentration are followed by a reduction in insomnia and its consequences, rather than the improvement of depressive symptoms [28].

Low levels of BDNF in depressive patients are also linked to relationship between stress and depression. Sleep disturbances decrease BDNF levels and the loss of sleep results in high vulnerability to stress and consequently leads to decrease in BDNF levels [15]. Stress is a potent risk factor for depression and is associated with decreased BDNF concentration in animal models. This hypothesis is since stress activates the Hypothalamic-pituitary-adrenal (HPA) axis leading to increased cortisol secretion and elevated cortisol concentrations could suppress the production of BDNF [16]. Stress reduces the expression of BDNF in the hippocampus and this reduction can be prevented by treatment with antidepressant drugs [3, 37].

If we look at all the exposed characteristics, it’s possible to observe that there is a chain of events that links sleep to depression. The loss of sleep quality (not necessarily a sleep disorders) leads to a high stress that suppresses BDNF secretion and consequently leads to great vulnerability to depressive conditions. One study showed that sleep is a mediator in the relationship between stress and BDNF, and that sleep disturbance would be a delimiter between people who deal well with stress and those who get sick [27]. In this sense, it is not possible to state that there is a causal pattern about sleep deprivation, BDNF and depression, but carefully examining all the studies in the field we can hypothesize it.

## 5 FUTURE PERSPECTIVES AND FINAL CONSIDERATIONS

Depression is nowadays the most common psychiatric disorder and is associated with high levels of disability [38]. Thereby, as sleep impairment is the main residual symptom of depression and a major risk factor for relapse, treatments that increase BDNF levels and consequently improve sleep quality will not only allow to improve depressive symptoms but also decrease relapse rates. Increasing the levels of this neurotrophin seems to improve the quality of sleep, allowing reducing vulnerability to stress and to create a protective factor to depression [16]. Furthermore, increasing our knowledge to deeply understand the role of BDNF in depression will allow developing more effective pharmacological agents to treat this disorder.

Further studies are necessary to clearly understand how BDNF levels influence and are influenced by depression and sleep deprivation. Moreover, alternative treatments that increase BDNF levels may also help to address the needs of non-depressive patients who also experience sleep impairment [39]. Finally, researchers should also make efforts to define the relationship between BDNF and sleep, as improving the quality of sleep may be a crucial step to improve patient’s quality of life [40, 41].

## Figures and Tables

**Table 1:** Results on the relationship between BDNF and sleep.

Authors	Aim	Sample	Methods	Instruments	Results
Rethorst *et al.* [19]	Examine biomarkers associated with changes in hypersomnia and insomnia.	126 Individuals with MDD.	Individuals were randomly assigned to two groups of aerobic exercise.	Inventory of Depressive Symptomatology (IDS-C). Blood Analysis.	Reduction of BDNF levels associated with decreased hypersomnia.
Giese *et al.* [31]	Investigate the serum BDNF level in adults with insomnia and compare them to a control group.	19 Individuals with RLS/PLM 7 patients with RLS 24 controls	BDNF levels were collected and correlated with the scores reported in the Insomnia Severity Index.	Insomnia Severity Index. Blood Analisys.	Insomniacs exhibit a significantly lower serum BDNF level than controls.
Rusch *et al.* [32]	Determine the relationship between increased sleep quality and improvement of depressive symptoms.	44 Individuals with insônia.	Subjects underwent a clinical evaluation and blood samples were taken from all participants. Participants were classified into two groups: sleep improved (n = 28) and sleep declined (n = 16). Participants underwent 4-8 sessions of CBT for insomnia (CBT-I).	Health-Related Quality of Life (HRQOL) Pittsburgh Sleep Quality Index (PSQI) Blood Analisys	The promotion of sleep quality is an effective way to improve depression and quality of life.
Giese *et al.* [30]	Investigate whether the level of stress influences the association of sleep and BDNF levels.	7 Individuals without insomnia and with RLS/PLM 24 controls 11 Individuals with subclinical insomnia and with RLS/PLM 8 Individuals with insomnia and with RLS.	Patients underwent clinical evaluation where the data were correlated.	Insomnia Severity Index (ISI) Perceived Stress Scale (PSS)	Sleep is a mediator in the relationship stress and BDNF. Sleep disturbance may explain how some people cope well with stress and other people get sick.

**Note:** MDD = Major Depressive Disorder; RLS = restless legs syndrome; PLM = periodic limb movement.

**Table 2:** Results on the relationship between BDNF and depression.

**Authors**	**Aim**	**Sample**	**Methods**	**Instruments**	**Results**
Shimizu *et al.* [10]	Determine how BDNF levels are related to treated and untreated depressive conditions and how these levels differ between individuals with MDD and controls.	16 individuals with MDD without antidepressant treatment. 17 individuals with MDD on antidepressant therapy. 50 controls.	Patients were assessed using the Hamilton Scale for depression (HAM-D). BDNF levels were accessed through the ELISA method.	Hamilton Depression Rating Scale (HAM-D)	Low levels of BDNF were found in untreated depressive patients when compared to the treatment group and the control group.
Siuciak *et al.* [34]	Determine how the administration of BDNF can produce an antidepressant effect in two animal models of depression.	Male rats treated in the laboratory.	Infusion of BDNF into the animals' brains for a week.	-	An antidepressant effect was observed after administration of BDNF in the two animal models of depression tested in the study. The increase could be mediated by increased activity in monoaminergic systems.
Martinotti *et al.* [5]	Investigate the effects of Agomelatine on serum BDNF levels in a sample of depressed patients.	27 individuals with MDD.	Serum levels of BDNF were achieved by the ELISA method at the beginning of treatment after two weeks and after 8 weeks of treatment.	Hamilton Depression Rating Scale (HAM-D). Snaith-Hamilton Pleasure Scale.	Patients showed an increase in BDNF levels after two weeks of treatment with Agomelatine.

**Note:** MDD = Major Depressive Disorder.
